# Beyond Nodal Stage: A Clinical Study of Colorectal Cancer Outcomes at a Single Tertiary Care Center

**DOI:** 10.7759/cureus.84365

**Published:** 2025-05-18

**Authors:** Seshaan KNB, Ruthrendhra Ethirajulu, Srinath Ganesan, Gouthaman S, Ganesh Guru

**Affiliations:** 1 General Surgery, Sree Balaji Medical College and Hospital, Chennai, IND; 2 Surgical Oncology, Good Samaritan Cancer Hospital, Eluru, IND; 3 Surgical Oncology, Sree Balaji Medical College and Hospital, Chennai, IND; 4 Surgical Oncology, Sri Ramachandra Institute of Higher Education and Research, Chennai, IND

**Keywords:** clavien-dindo score, colon resection, colorectal cancer, lymph node ratio, perineural invasion

## Abstract

Introduction

Colorectal cancer (CRC) continues to be one of the most frequently diagnosed cancers worldwide and a major contributor to cancer-related mortality. The ratio of the number of involved to the total number of resected lymph nodes is termed as lymph node ratio (LNR), which is an important prognostic factor in colorectal malignancies.

Aim

This study aims to evaluate the prognostic value of lymph node ratio (LNR) in colorectal cancer (CRC), with particular reference to its association with survival outcomes.

Materials and methods

A retrospective analysis of 46 patients who underwent surgery for CRC in a single tertiary care center from October 2015 to July 2019 was conducted. Data on demographic variables, tumor histology, treatment modalities, and survival outcomes were collected using a retrospective chart review. The chi-square test was used to analyze the variables. All data were recorded using a standard data form and analyzed using SPSS version 21.0 (IBM Corp., Armonk, NY). Statistical significance was defined as p<0.05. We acknowledge that the study is based on a relatively small sample from a single institution, which may limit its statistical power and introduce potential selection bias. However, the findings offer meaningful preliminary data regarding LNR as a prognostic marker in CRC and highlight the need for validation through larger, multicenter studies.

Results

The mean age of the study population is 56 years. The rectum was the primary cancer site involved in 25 patients (54.3%). The most common pathology was adenocarcinoma in 32 (69%) patients. Lymphovascular and perineural invasion (PNI) was present in 12 (26%) and three (6.5%) patients, respectively. Eighteen patients belonged to the T2 (39%) stage, and 20 patients (60%) had a nodal status of N0. Most patients experienced Grade II postoperative complications (n=37, 80.4%), while Grade III and IV complications were observed in seven (15.2%) and two (4.3%) patients, respectively. Fifteen patients (32%) were given treatment prior to surgery in the form of neoadjuvant chemoradiation. The mean survival period in our study is 50 months. The presence of perineural invasion (p<0.019), node positivity (p<0.005), CD score of more than or equal to 3 (p<0.001), and higher lymph node ratio (p<0.001) were determined as independent prognostic factors for survival (p<0.05).

Conclusion

Lymph node ratio is a powerful factor for estimating the survival of CRC patients. Good postoperative care and recovery with a low CD score and meticulous surgery with higher lymph node yield would alter the survival status in CRC patients.

## Introduction

Colorectal cancer (CRC) stands as one of the most common and lethal malignancies worldwide. It ranks as the third most frequently diagnosed cancer and the fourth leading cause of cancer-related deaths globally. In recent decades, many Asian nations, including Japan and Singapore, have witnessed a twofold to fourfold surge in colorectal cancer incidence. This rise is largely attributed to factors such as population aging, sedentary lifestyles, smoking, poor dietary habits, and increasing rates of obesity [1].

The prognosis of colorectal cancer is heavily influenced by several clinicopathological factors that determine the biological behavior of the tumor, its response to treatment, and the long-term outcomes. Over the years, various studies have explored the prognostic implications of clinical characteristics, histological features, and demographic profiles [2]. Among the emerging prognostic markers, the lymph node ratio (LNR), defined as the ratio of metastatic lymph nodes to the total number of resected nodes, has gained significant attention. It is increasingly recognized as a more reliable indicator of patient survival compared to the absolute number of positive lymph nodes alone [3].

With improved understanding of prognostic parameters and the integration of structured follow-up protocols, it has become possible to enhance early detection and optimize therapeutic decision-making. This study was therefore designed to identify key prognostic factors in colorectal cancer and to evaluate their relationship with overall survival. In particular, emphasis was placed on the role of LNR and its correlation with postoperative complications, pathological parameters, and long-term outcomes using a comprehensive survival analysis [4].

## Materials and methods

This study included a total of 46 patients diagnosed with colorectal cancer. The cohort showed a male predominance, with 31 men (67.4%) and 15 women (32.6%). The mean age of the patients was 56 years. The most common tumor location was the rectum, seen in 28 patients (60.8%), followed by the left colon in 10 patients (21.7%) and the right colon in eight patients (17.4%). Histopathological analysis revealed that adenocarcinoma was the most frequent subtype, present in 32 cases (69.5%), while mucinous carcinoma was identified in 11 cases (23.9%). Other histological types accounted for three cases (6.5%). Regarding tumor grade, a majority of patients had high-grade tumors (71.7%), while the remaining 28.3% had moderate-grade tumors. Perineural invasion (PNI) was observed in three patients (6.5%), suggesting a potential for aggressive tumor behavior in a small subset of the population, as shown in Table 1.

**Table 1:** Clinicopathological Characteristics of the Study Population (n=46)

Variable	Number of Patients (%)
Gender	
Male	31 (67.4%)
Female	15 (32.6%)
Mean age (years)	56
Tumor location	
Rectum	28 (60.8%)
Right colon	8 (17.4%)
Left colon	10 (21.7%)
Histopathology	
Adenocarcinoma	32 (69.5%)
Mucinous carcinoma	11 (23.9%)
Others	3 (6.5%)
Tumor grade	
High grade	33 (71.7%)
Moderate grade	13 (28.3%)
Perineural invasion	3 (6.5%)

Among the 46 patients, the majority had locally advanced tumors. T2 stage was seen in 18 (39.1%) and T3 in 17 (37%), while early-stage T1 was observed in one (2.2%). Advanced T4 disease was noted in seven (15.2%). A complete pathological response following neoadjuvant treatment was achieved in three rectal cancer patients (10.7%). Regarding nodal status, 28 patients (60.9%) were node-negative (N0), while 10 (21.7%) had N1 and eight (17.4%) had N2 nodal involvement. The lymph node ratio (LNR) was zero in 31 patients (67.4%), indicating no positive nodes, while 15 (32.6%) had a ratio greater than zero. The average number of lymph nodes harvested during surgery was 15. Postoperative outcomes were assessed using the Clavien-Dindo (CD) classification. Most patients had Grade II complications (n=37, 80.4%), while more severe complications, Grades III and IV, were seen in seven (15.2%) and two (4.3%) patients, respectively. The mean survival time for the cohort was 50 months, as shown in Table 2.

**Table 2:** Pathological and Survival Variables CD: Clavien-Dindo

Variable	Number of Patients n (%)
T stage	
T1	1 (2.2%)
T2	18 (39.1%)
T3	17 (37%)
T4	7 (15.2%)
Complete response (rectal)	3 (10.7%)
Nodal stage	
N0	28 (60.9%)
N1	10 (21.7%)
N2	8 (17.4%)
Lymph node ratio (LNR)	
0 (no positive nodes)	31 (67.4%)
>0 (positive nodes present)	15 (32.6%)
Mean nodes harvested	15
Postoperative CD score	
II	37 (80.4%)
III	7 (15.2%)
IV	2 (4.3%)
Mean survival time (months)	50

**Figure 1:**
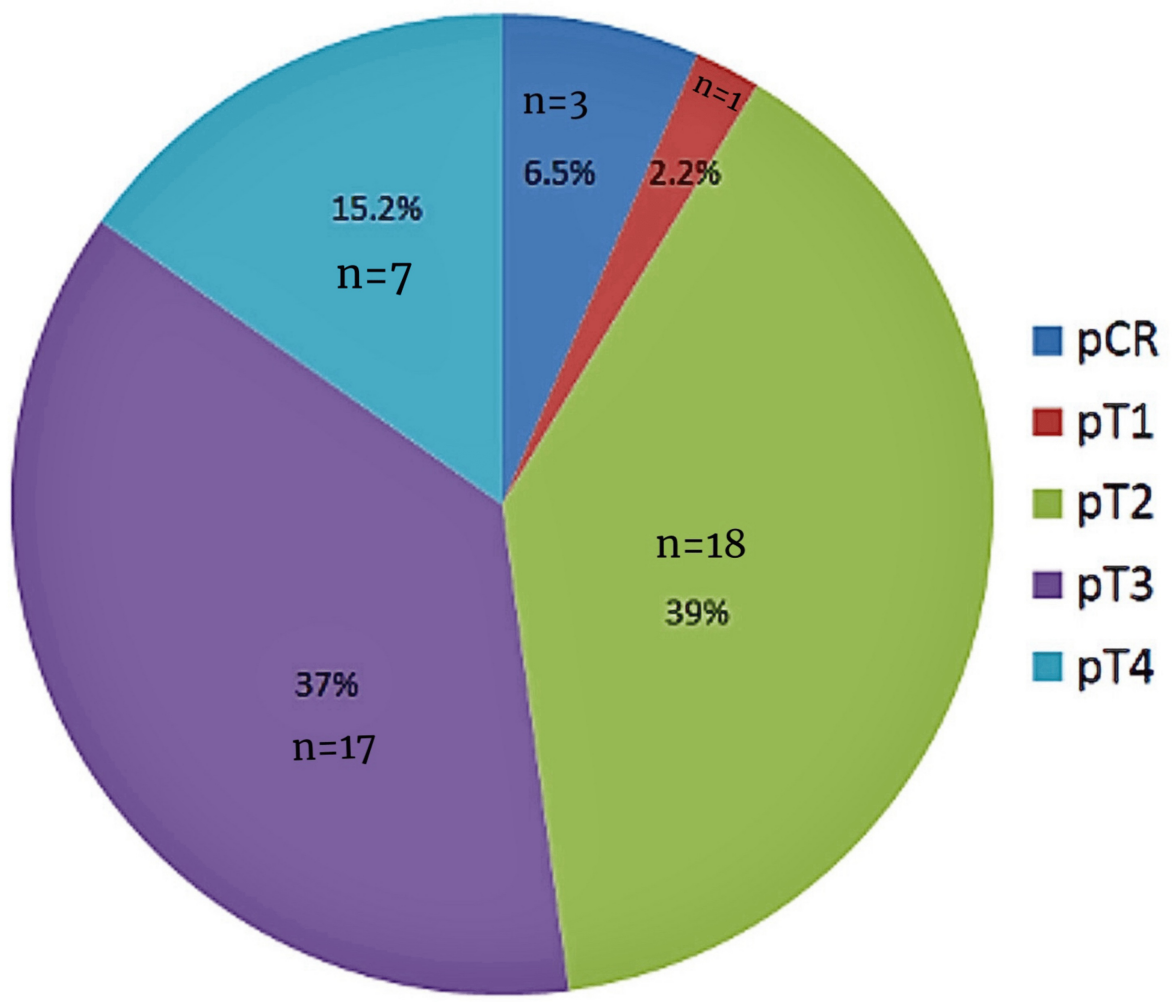
Distribution of Tumor (T) Stages Among Patients Post Surgery pCR, pathological complete response; pT, pathological T staging

This pie chart (Figure 1) depicts the pathological T staging (pT) of the tumors in the study cohort. The most common stage was pT2, observed in 39% of patients (n=18), followed closely by pT3 in 37% (n=17). Advanced tumors classified as pT4 accounted for 15.2% (n=7), while early-stage pT1 was noted in only one patient (2.2%). Additionally, a pathological complete response (pCR) following neoadjuvant therapy was seen in 6.5% of patients (n=3), indicating the absence of residual tumor in the resected specimen. This distribution suggests a predominance of intermediate-stage disease in the studied population.

**Figure 2:**
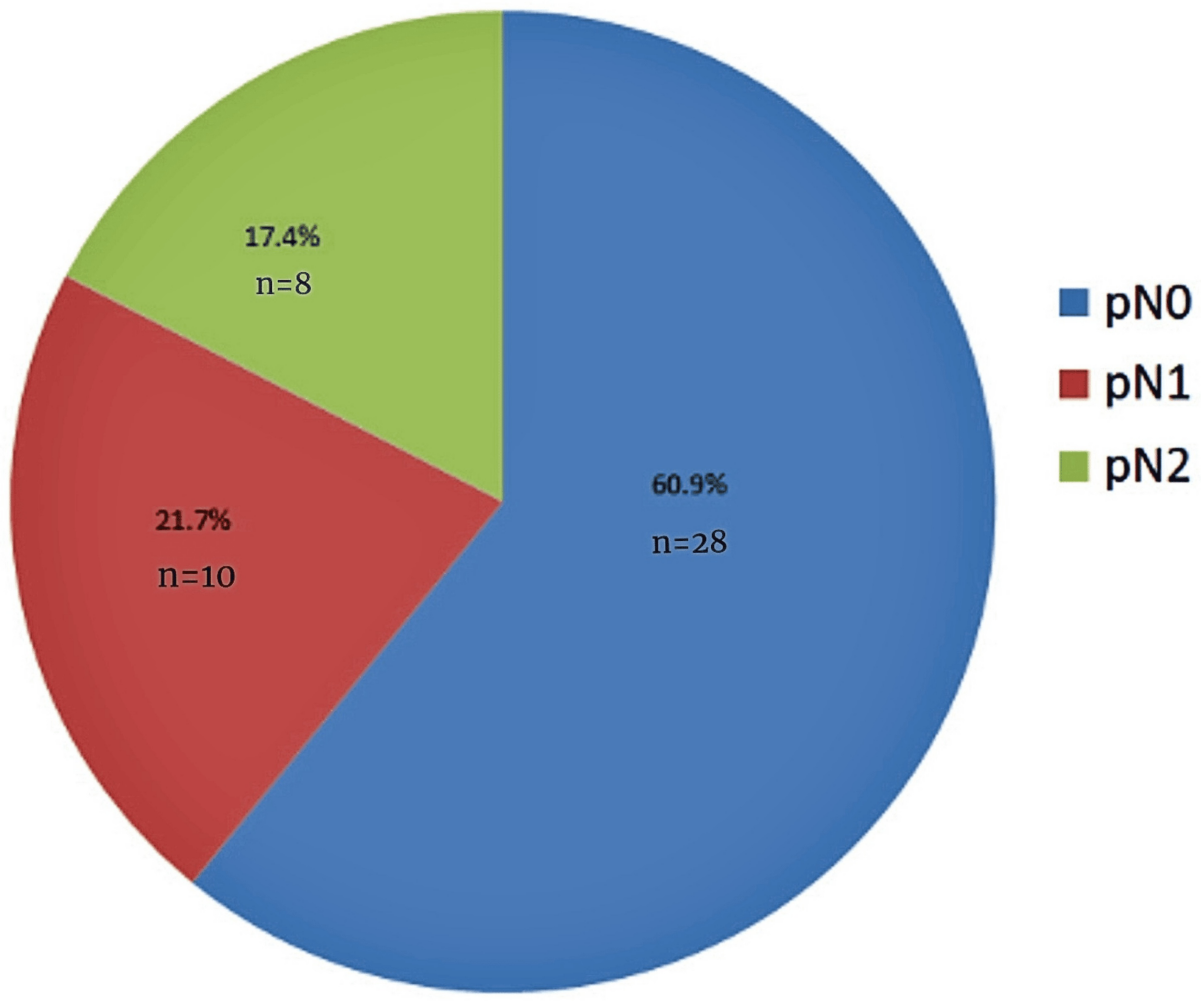
Distribution of Nodal (N) Stages in Resected Colorectal Cancer Specimens pN: pathological nodal staging

The pie chart (Figure 2) illustrates the pathological nodal staging (pN) of the 46 patients included in the study. A majority of patients (60.9%, n=28) were classified as pN0, indicating no regional lymph node metastasis. pN1 nodal involvement (1-3 positive lymph nodes) was observed in 21.7% of patients (n=10), while pN2 stage (≥4 positive lymph nodes) was identified in 17.4% (n=8). This distribution highlights that most patients were node-negative at the time of surgery.

## Results

A total of 46 patients with histologically confirmed colorectal cancer (CRC) were included in this study. The mean age of the patients was 56 years, with a predominance of men (n=31, 67.4%) compared to women (n=15, 32.6%). The rectum was the most frequently involved site (n=28, 60.8%), followed by the left colon (n=10, 21.7%) and the right colon (n=8, 17.4%). The most common histological subtype was adenocarcinoma (n=32, 69.5%), followed by mucinous carcinoma (n=11, 23.9%) and other subtypes (n=3, 6.5%) (Table 3).

**Table 3:** Patient Demographics and Clinical Features

Characteristic	Value, n (%)
Total number of patients	46
Mean age (years)	56
Gender	
Male	31 (67.4%)
Female	15 (32.6%)
Tumor location	
Rectum	28 (60.8%)
Right colon	8 (17.4%)
Left colon	10 (21.7%)
Histopathology	
Adenocarcinoma	32 (69.5%)
Mucinous carcinoma	11 (23.9%)
Others	3 (6.5%)

Among patients with rectal cancer (n=28), 50% (n=14) received neoadjuvant chemoradiation. Additionally, one patient with colon cancer (5.5%) received neoadjuvant chemotherapy alone. Tumor staging revealed T2 disease in 18 patients (39.1%) and T3 disease in 17 patients (37%), while T1 and T4 stages were observed in one (2.2%) and seven (15.2%) patients, respectively. A complete pathological response following neoadjuvant therapy was seen in three rectal cancer patients (10.7%). The mean number of lymph nodes harvested was 15. Node-negative status (N0) was present in 28 patients (60.9%), and a lymph node ratio (LNR) of zero was identified in 31 patients (67.9%) (Table 4). Although the lymph node ratio (LNR) was significantly associated with overall survival, a specific cutoff value to stratify high-risk patients could not be determined in this study due to the limited sample size. Among patients, 31 (67.9%) had an LNR of zero, while the remaining 15 (32.1%) had varying degrees of nodal involvement. The limited distribution of LNR values restricted the ability to perform reliable statistical modeling to define a clinically meaningful threshold.

**Table 4:** Pathological and Treatment Characteristics pT: pathological T staging

Characteristic	Value
Tumor grade	
High grade	33 (71.7%)
Moderate grade	13 (28.3%)
Tumor stage (pT)	
T1	1 (2.2%)
T2	18 (39.1%)
T3	17 (37%)
T4	7 (15.2%)
Complete pathological response (rectal)	3 (10.7%)
Lymphovascular invasion	12 (26%)
Perineural invasion	3 (6.5%)
Neoadjuvant treatment	
Rectal cancer (chemoradiation)	14 (50%)
Colon cancer (chemotherapy)	1 (5.5%)

Low anterior resection was the most common surgical procedure, performed in 11 patients (23%). The mean number of lymph nodes harvested was 15. Regarding nodal status, 28 patients (60.9%) were node-negative (N0), while 10 (21.7%) had N1, and eight (17.4%) had N2 disease. A lymph node ratio (LNR) of zero was observed in 31 patients (67.9%). Postoperative complications, graded using the Clavien-Dindo (CD) classification, showed that 37 patients (80.4%) had Grade II complications, seven (15.2%) had Grade III, and two (4.3%) had Grade IV complications. The mean survival duration was 50 months. On statistical analysis, a high LNR (p<0.001), node positivity (p<0.005), CD score of ≥3 (p<0.001), and the presence of perineural invasion (p<0.019) were identified as significant independent prognostic factors associated with reduced survival (Table 5).

**Table 5:** Surgical and Survival Outcomes CD: Clavien-Dindo

Characteristic	Value
Most common surgery	Low anterior resection (11, 23%)
Mean number of nodes harvested	15
Nodal status	
N0	28 (60.9%)
N1	10 (21.7%)
N2	8 (17.4%)
Lymph node ratio=0	31 (67.9%)
Postoperative CD score	
Grade II	37 (80.4%)
Grade III	7 (15.2%)
Grade IV	2 (4.3%)
Mean survival duration	50 months

Several prognostic factors were found to have a statistically significant association with overall survival in this study. A high lymph node ratio (LNR) was strongly correlated with poor prognosis (p<0.001), reinforcing its role as an independent marker of disease burden. Similarly, patients with Clavien-Dindo scores of 3 or higher experienced significantly worse outcomes (p<0.001), highlighting the impact of severe postoperative complications on long-term survival. Nodal positivity (p<0.005) and the presence of perineural invasion (p<0.019) were also associated with reduced survival, suggesting more aggressive disease behavior. These findings emphasize the importance of comprehensive pathological and postoperative assessment, as shown in Table 6.

**Table 6:** Prognostic Factors Significantly Associated With Reduced Survival CD: Clavien-Dindo

Prognostic Factor	P-value	Significant
High lymph node ratio	<0.001	Yes
CD score of ≥3	<0.001	Yes
Node positivity	<0.005	Yes
Perineural invasion	<0.019	Yes

**Figure 3:**
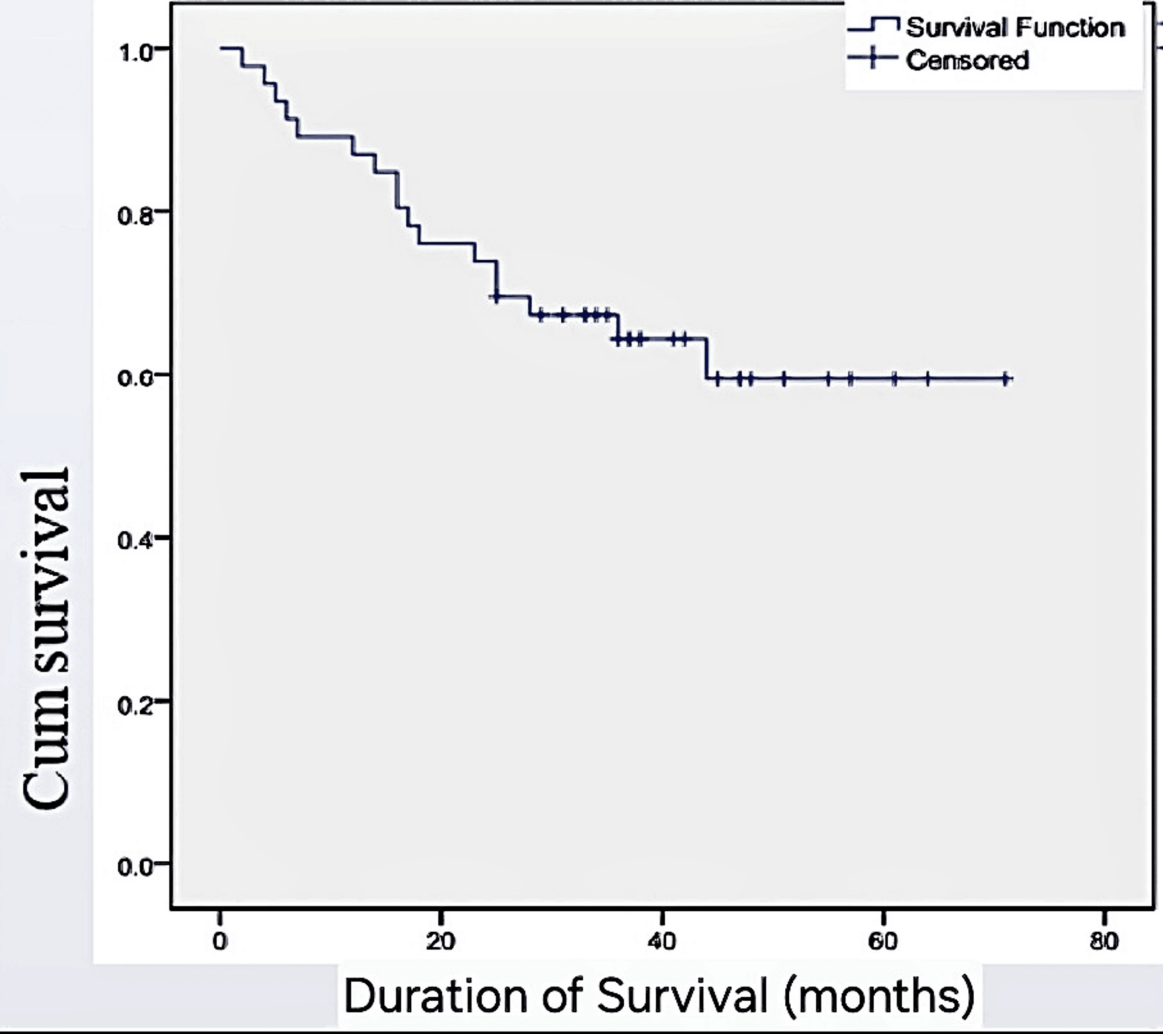
Overall Survival Curve of the Study Population Cum: cumulative

This Kaplan-Meier survival curve depicts the overall survival of the study cohort. The mean survival period was found to be 50 months. The graph emphasizes the long-term survival trends among patients who underwent surgery for colorectal cancer, with various clinicopathological factors impacting survival duration (Figure 3).

**Figure 4:**
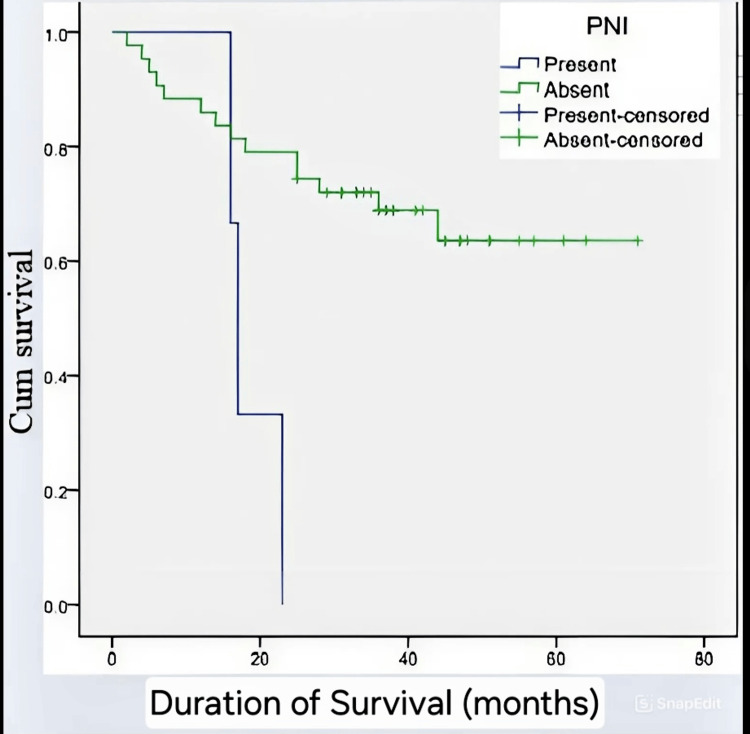
Impact of Perineural Invasion (PNI) on Overall Survival Cum: cumulative

This Kaplan-Meier curve evaluates the effect of perineural invasion (PNI) on overall survival. Patients with PNI-positive tumors had notably worse survival compared to PNI-negative cases (p<0.019). The presence of PNI was identified as a strong independent prognostic factor for reduced survival outcomes in colorectal cancer (Figure 4).

**Figure 5:**
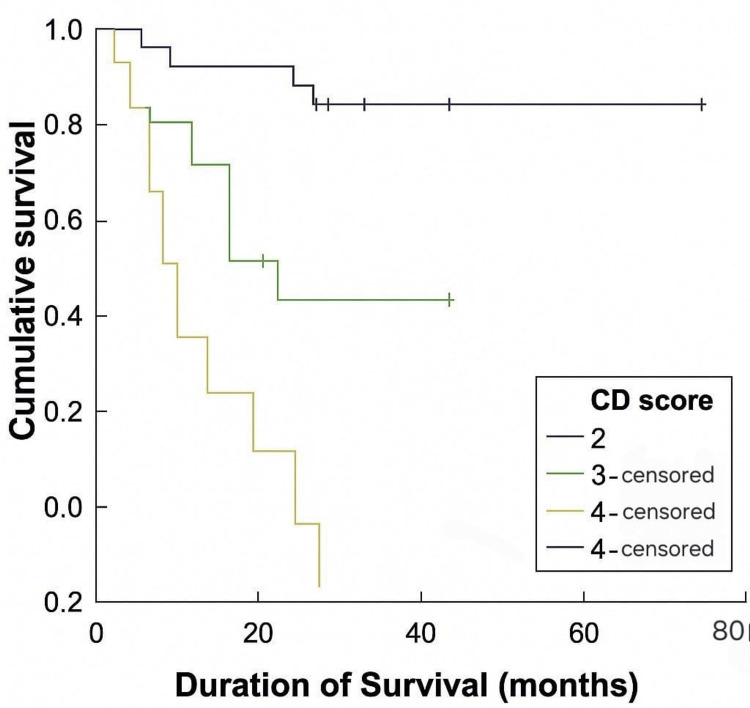
Survival Analysis Based on Postoperative Clavien-Dindo (CD) Scores

This Kaplan-Meier plot analyzes survival outcomes according to postoperative Clavien-Dindo (CD) complication scores. Patients with CD scores of 2 had significantly better survival compared to those with scores of ≥3 (p<0.001). Postoperative complication severity emerged as an independent prognostic factor impacting long-term survival in colorectal cancer patients (Figure 5).

## Discussion

Colorectal cancer is increasingly recognized as a heterogeneous disease in the era of precision medicine, with tumors classified by anatomical location into right-sided colon, left-sided colon, and rectal sub-sites (upper, mid, and lower rectum). This distinction is clinically relevant, as tumor biology, lymphatic drainage patterns, surgical approaches, and response to therapy differ significantly among these locations. It is well established that right-sided tumors are associated with higher lymph node yields and often different molecular profiles, whereas rectal cancers have distinct staging and treatment paradigms. Given this variability, lymph node involvement and consequently the lymph node ratio (LNR) may have different prognostic implications based on tumor location.

In our study, due to the small sample size, we were unable to perform a reliable sub-analysis based on tumor location. We recognize this as a key limitation. Stratifying patients according to tumor sub-site could provide a more nuanced understanding of the prognostic relevance of LNR in each anatomical region. Future studies with larger, multi-institutional datasets should aim to evaluate LNR across these subgroups to improve prognostic accuracy and guide individualized treatment decisions.

Surgical resection continues to be the foundation of treatment for nonmetastatic colorectal cancer. The success of this intervention is dependent not only on tumor characteristics but also on the quality of surgical technique, the accuracy of preoperative staging, and adherence to standardized treatment protocols. Patient-related factors such as age, physical fitness, perioperative care, and tumor staging further influence surgical outcomes and long-term survival [5].

This study has several limitations. The small sample size (46 patients) limits generalizability, and the single-center design may not reflect outcomes in broader settings. Additionally, the retrospective nature of some data collection and reliance on historical records could introduce biases. The study did not assess molecular factors or patient-reported outcomes, which are important for comprehensive survival analysis. Lastly, the follow-up period, with a mean survival of 50 months, may not capture long-term outcomes, especially for patients with advanced disease. Larger, multicenter studies with longer follow-up are needed to validate these findings.

In this study, several prognostic markers were found to significantly affect overall survival. Among them, lymph node positivity demonstrated a strong correlation with poorer prognosis. The number of positive lymph nodes, analyzed through univariate statistics, emerged as an independent predictor of reduced survival. This reinforces the importance of meticulous nodal dissection and comprehensive pathological evaluation in colorectal cancer surgeries [4,5].

Neoadjuvant therapy played a significant role in the subset of rectal cancer patients, where it contributed to tumor downstaging and potentially increased the chances of achieving complete pathological response. In the current cohort, half of the rectal cancer patients received neoadjuvant chemoradiation [6]. This approach likely contributed to better surgical margins and improved local control.

Postoperative complications, particularly those of higher severity as graded by the Clavien-Dindo classification, were associated with unfavorable oncological outcomes. Patients who experienced complications of Grade III or higher had significantly shorter survival periods, highlighting the need for optimized perioperative protocols [7,8]. The adoption of enhanced recovery strategies such as early mobilization, prehabilitation, and nutritional optimization can help reduce complication rates and improve recovery trajectories.

The lymph node ratio (LNR) emerged as a statistically significant prognostic factor in this study. Patients with lower LNRs demonstrated superior survival outcomes compared to those with higher values, independent of the total number of nodes retrieved. This observation reinforces the prognostic advantage of assessing not only the presence of nodal metastasis but also the relative burden of disease, as captured by LNR [9,10]. While a defined threshold such as LNR of >0.4 has shown prognostic relevance in prior studies, the small sample size in our cohort limited our ability to establish an optimal cutoff. Larger, multicentric datasets would be necessary to validate such a threshold and assess its integration into staging systems.

Beyond its prognostic relevance, LNR may also have future utility in therapeutic decision-making. For example, patients with stage II disease but a high LNR, reflecting extensive nodal involvement relative to yield, may benefit from adjuvant chemotherapy even in the absence of classic high-risk features [11]. Additionally, LNR could be incorporated into postoperative risk stratification models to tailor surveillance protocols, identify candidates for clinical trials, or adjust treatment intensity. Its integration into individualized care algorithms represents an important area for future research [12].

In parallel, the presence of perineural invasion (PNI) was another histopathological marker independently associated with poorer outcomes [12,13]. PNI, indicative of aggressive tumor biology, remained significant even when adjusted for stage and nodal status. The thorough histological evaluation in this study, which included the assessment of tumor grade, margins, lymphovascular invasion, and PNI, emphasizes the need for comprehensive reporting to ensure accurate prognostication [14,15]. The average lymph node yield in our study met oncological standards, supporting the adequacy of surgical and pathological evaluation.

Taken together, these findings underscore the multifactorial determinants of prognosis in colorectal cancer [15]. Incorporating both quantitative and qualitative pathological factors, such as LNR and PNI, into routine clinical assessment could enhance survival prediction and inform individualized treatment strategies moving forward.

## Conclusions

LNR is a powerful, independent predictor of survival in colorectal cancer. Together with PNI and postoperative CD score, it offers a more nuanced understanding of prognosis beyond the traditional tumor, node, and metastasis (TNM) system. Strategies to optimize node harvest, reduce surgical complications, and tailor adjuvant therapies can substantially improve outcomes. This retrospective analysis suggests that lymph node ratio (LNR) may serve as a useful prognostic indicator in colorectal cancer patients, especially when evaluated alongside established factors such as nodal status, perineural invasion, and postoperative complication severity. While our findings highlight an association between higher LNR and reduced survival, the study’s limited sample size and population heterogeneity warrant cautious interpretation. Larger, prospective studies with site-specific stratification are needed to validate the prognostic utility of LNR and to refine risk stratification in colorectal cancer.
